# Supplementary material for: Inhibition of c-Jun in AgRP neurons increases stress-induced anxiety and colitis susceptibility
Source: Commun Biol. 2023 Jan 14;6:50. doi: 10.1038/s42003-023-04425-w (PMC9840628; doi:10.1038/s42003-023-04425-w)
Supplement: Supplementary file 2 — Supplementary information-clean [file 42003_2023_4425_MOESM2_ESM.pdf]

Supplementary information

Supplementary Figures and Figure Legends

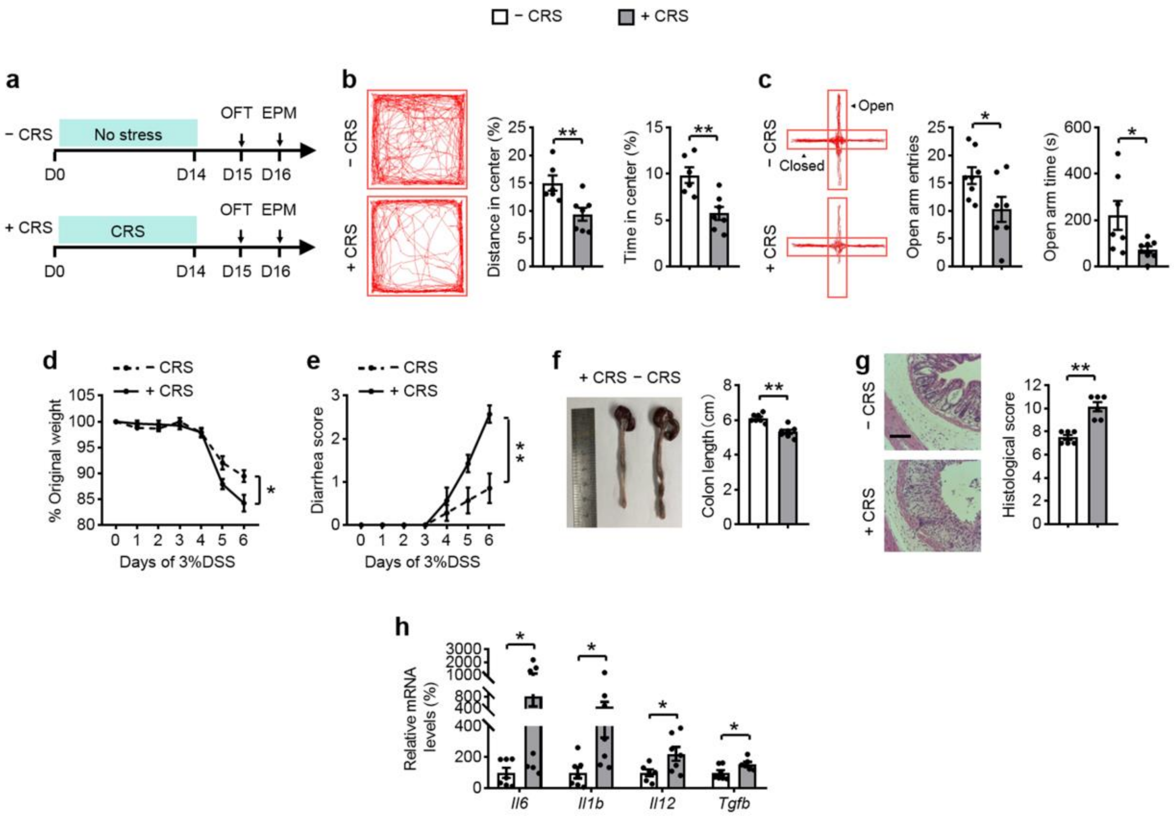

Supplementary Figure 1. Chronic restraint stress (CRS) induces anxiety-like behaviors and increases the susceptibility to colitis. (**a**) Schematic showing the CRS experimental protocol. (**b**) Representative tracks and statistical results in OF test. (**c**) Representative tracks and statistics in EPM test. (**d**) Percentage of body weight loss. (**e**) Scores of diarrhea. (**f**) Gross morphology and length of the colon. (**g**) H&E staining and histological scores of the colon tissues. Scale bar, 110 µm. (**h**) qRT-PCR analysis of mRNA expression of inflammatory cytokines (*Il6, Il1b, Il12*, and *Tgfb*) in the distal colon tissues. Studies for **a-c** were conducted using 12-week-old WT mice with unstress (- CRS) or stressed (+ CRS) treatment for 14 days. Behavioral tests

were performed on day 15 (**b**) and day 16 (**c**). **d-h** were conducted using - CRS mice and + CRS mice with 3% DSS in drinking water for 6 days to induce acute colitis; Values are expressed as means ± SEM (n = 6–8 per group), with individual data points. Data were analyzed using two-tailed unpaired Student's *t* test (**b, c, f-h**). Data were analyzed using two-way ANOVA with Bonferroni's multiple comparisons test (**d, e**). **P* <0.05, ***P* <0.01.

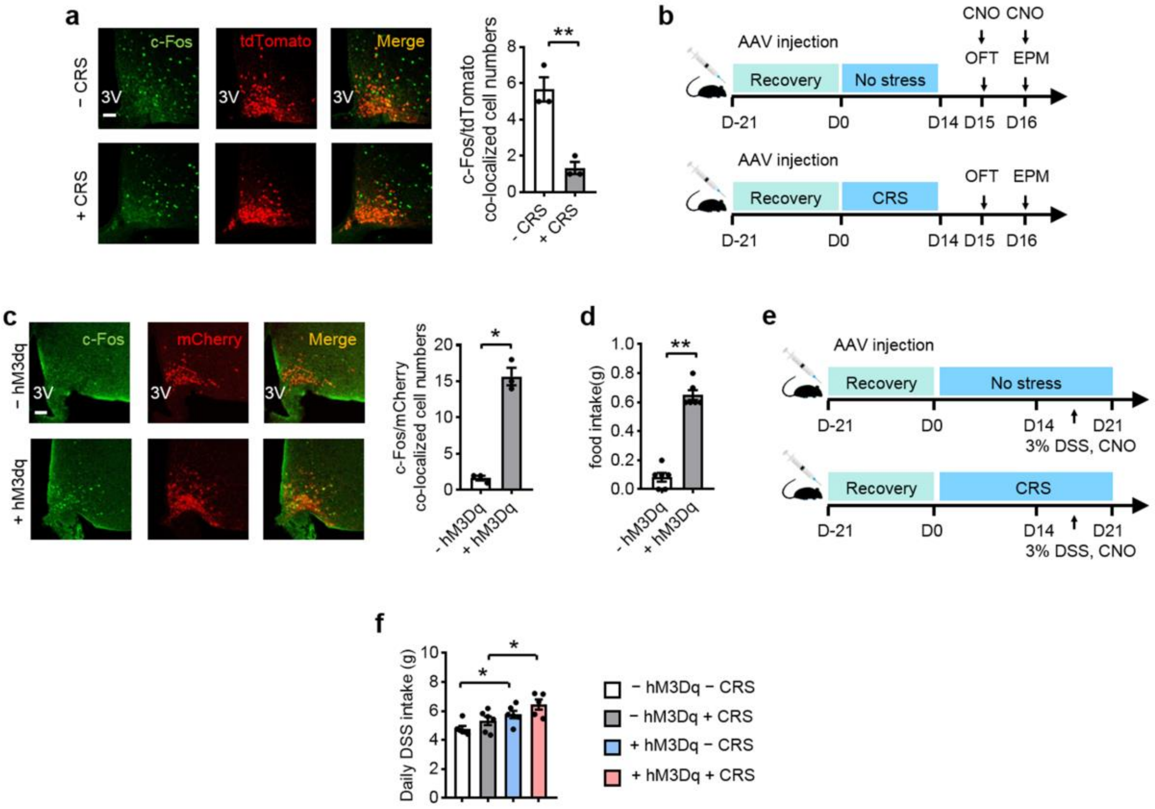

Supplementary Figure 2. Parameters related to mice with activation of AgRP neuronal activity. (**a**) Immunofluorescence (IF) staining for tdTomato (red), c-Fos (green), and merge (yellow) in the ARC sections (left), and quantification of c-Fos and tdTomato co-localized cell numbers (right). Scale bar, 50 μm. c-Fos staining was coupled with a TSA Plus Fluorescein KIT. (**b**) Experimental timeline for CRS. (**c**) IF

staining for mCherry (red), c-Fos (green) and merge (yellow) in ARC sections (left), and quantification of c-Fos and mCherry colocalized cell numbers (right). Scale bar, 50 μm. (**d**) Food intake. (**e**) Experimental timeline for DSS administration. (**f**) DSS intake. Study for **a** was conducted using 14-week-old *AgRP-Cre*-Ai9 mice with (+ CRS) or without (- CRS) 14 days of stress. **c-d** were conducted using 10- to 12-week-old *AgRP-Cre* mice receiving AAV expressing mCherry (- hM3Dq) or hM3Dq (+ hM3Dq), both treated with one injection of CNO and 30 min later for immunofluorescence analysis. Food intake was assessed 30 minutes after injection. **f** was performed using - hM3Dq mice and + hM3Dq mice under treatment of 3% DSS in drinking water for 7 days to induce acute colitis with (+ CRS) or without (- CRS) stress, simultaneously receiving CNO injections every 12 hours per day. Values are expressed as means ± SEM (n = 3-6 per group), with individual data points. Data were analyzed using two-tailed unpaired Student's *t* test (**a, c, d**). Data were analyzed using two-way ANOVA, followed by Tukey's multiple comparisons test (**f**). **P* <0.05, ***P* <0.01.

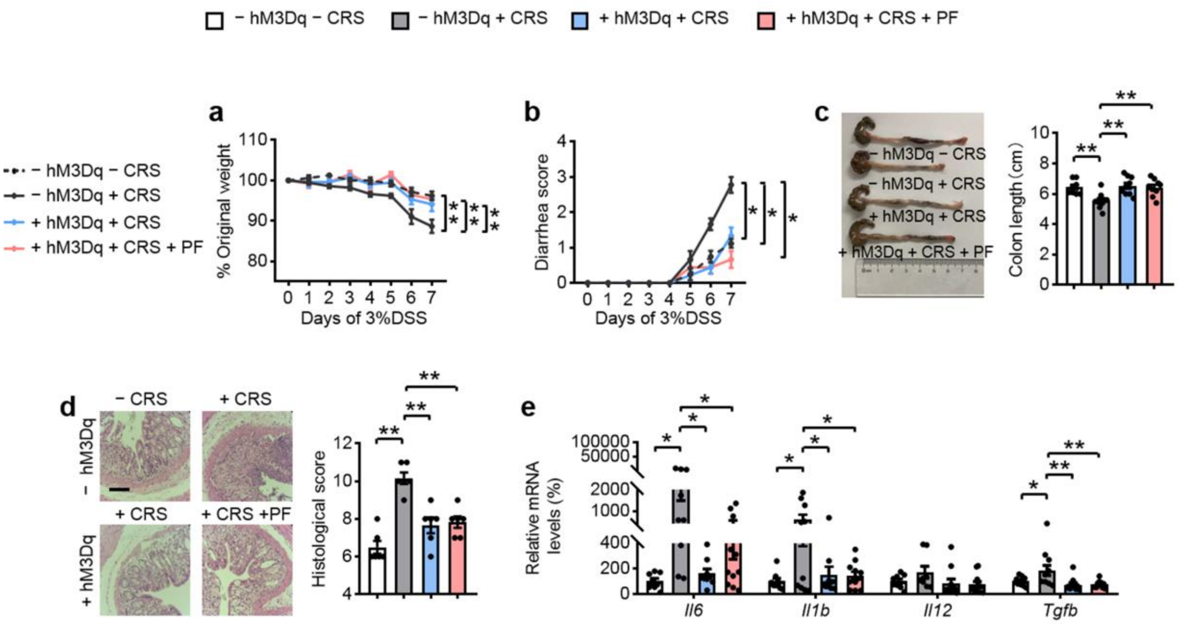

Supplementary Figure 3 Pair-feeding has no beneficial effect on the improvement of colitis. (**a**) Percentage of body weight loss. (**b**) Scores of diarrhea. (**c**) Gross morphology and length of the colon. (**d**) H&E staining and histological scores of the colon tissues. Scale bar, 110 μm. (**e**) qRT-PCR analysis of mRNA expression of inflammatory cytokines (*Il16*, *Il1b*, *Il12*, and *Tgfb*) in the distal colonic tissues. Studies were conducted using 10- to 12-week-old *AgRP-Cre* mice receiving AAV expressing mCherry (- hM3Dq) or hM3Dq (+ hM3Dq), all mice experienced unstressed (- CRS) or stressed (+ CRS) treatment for 14 days, after that was under treatment of 3% DSS in drinking water for 7 days to induce acute colitis, simultaneously receiving CNO injections every 12 hours per day. Pair-fed (PF) experiment was administered during CNO injection. For pair-fed groups (+ hM3Dq + CRS + PF), mice were given the same diet as the - hM3Dq + CRS groups. Values are expressed as means ± SEM (n=6-11 per group), with individual data points. Data were

analyzed using two-way ANOVA, followed by Tukey's multiple comparisons test. *P <0.05, **P <0.01.

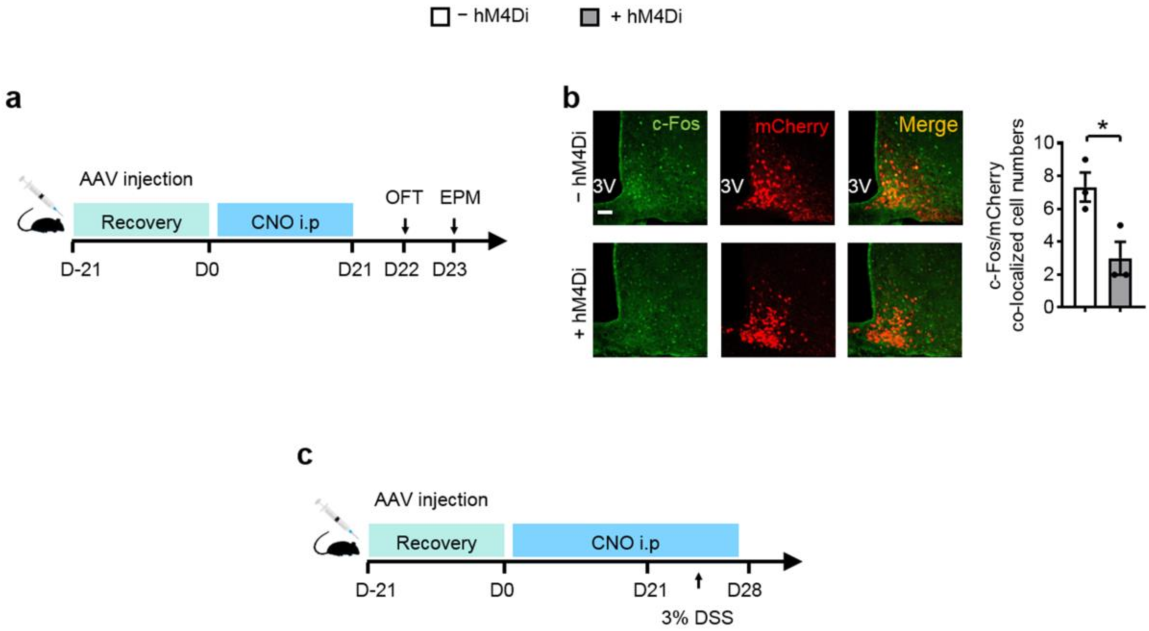

Supplementary Figure 4. Parameters related to mice with inhibition of AgRP neuronal activity. (**a**) Experimental timeline for CRS. (**b**) Immunofluorescence (IF) staining for mCherry (red), c-Fos (green) and merge (yellow) in ARC sections (left), and quantification of c-Fos and mCherry colocalized cell numbers (right). Scale bar, 50 µm. (**c**) Experimental timeline for DSS administration. Study for **b** was conducted using 10- to 12-week-old *AgRP-Cre* mice receiving AAV expressing mCherry (- hM4Di) or hM4Di (+ hM4Di) both treated with one injection of CNO and 30 min later for immunofluorescence analysis. Values are expressed as means ± SEM (n = 3 per group), with individual data points. Data were analyzed using two-tailed unpaired Student's *t* test. *P <0.05.

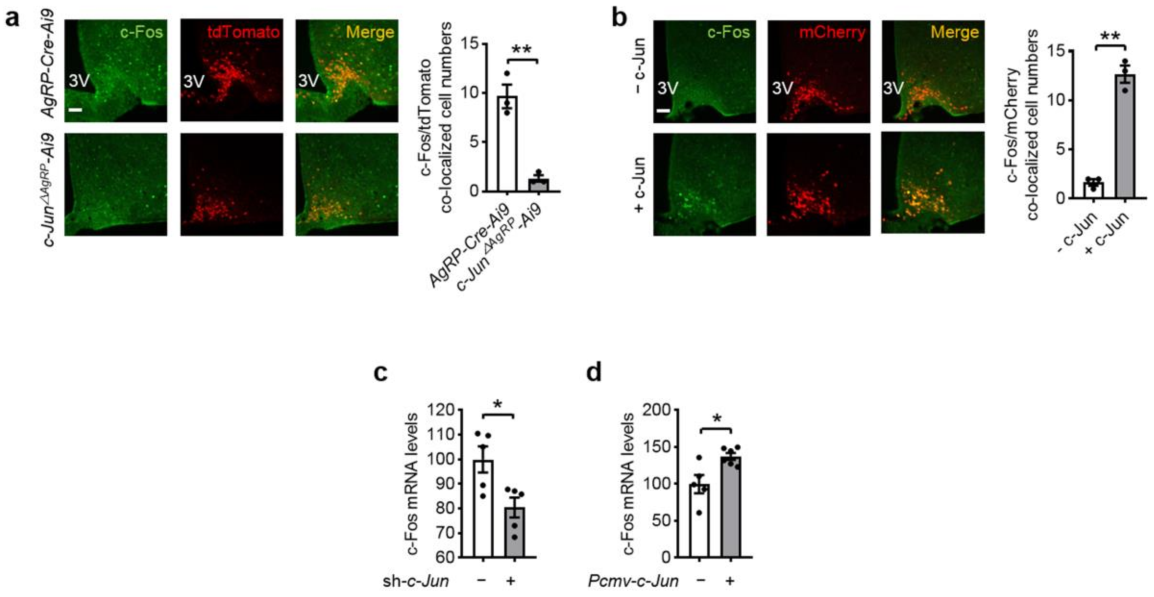

Supplementary Figure 5. The changes of c-Fos after c-Jun deletion and overexpression in vitro and in vivo. (**a**) IF staining for tdTomato (red), c-Fos (green) and merge (yellow) in ARC sections (left), and quantification of c-Fos and tdTomato colocalized cell numbers (right). Scale bar, 50 μm. (**b**) IF staining for mCherry (red), c-Fos (green) and merge (yellow) in ARC sections (left), and quantification of c-Fos and mCherry colocalized cell numbers (right). Scale bar, 50 μm. (**c, d**) C-Fos mRNA levels. Study for **a** was conducted by mating Ai9 (tdTomato) mice with *AgRP-irs-Cre* mice to obtain *AgRP*-Ai9 mice as controls, mating Ai9 (tdTomato) mice with mice with c-Jun deletion in AgRP neurons (*c-Jun*$^{\Delta AgRP}$) to obtain *c-Jun*$^{\Delta AgRP}$-Ai9 mice. **b** was conducted using 12- to 14-week-old *AgRP-Cre* mice receiving AAV expressing mCherry (- c-Jun) or c-Jun (+ c-Jun). **c** was conducted on primary hypothalamus isolated from newborn mice, receiving control (-sh-*cJun*) or sh-*cJun* (+ sh-*cJun*) transfected with Lipo 3000. **d** was conducted on primary hypothalamus isolated from

newborn mice, receiving control (- pcmv-*c-Jun*) or c-Jun (+ pcmv-*c-Jun*) transfected with Lipo 3000. Values are expressed as means ± SEM (n=3-6 per group), with individual data points. Data were analyzed using two-tailed unpaired Student's *t* test. **P* <0.05, ***P* <0.01.

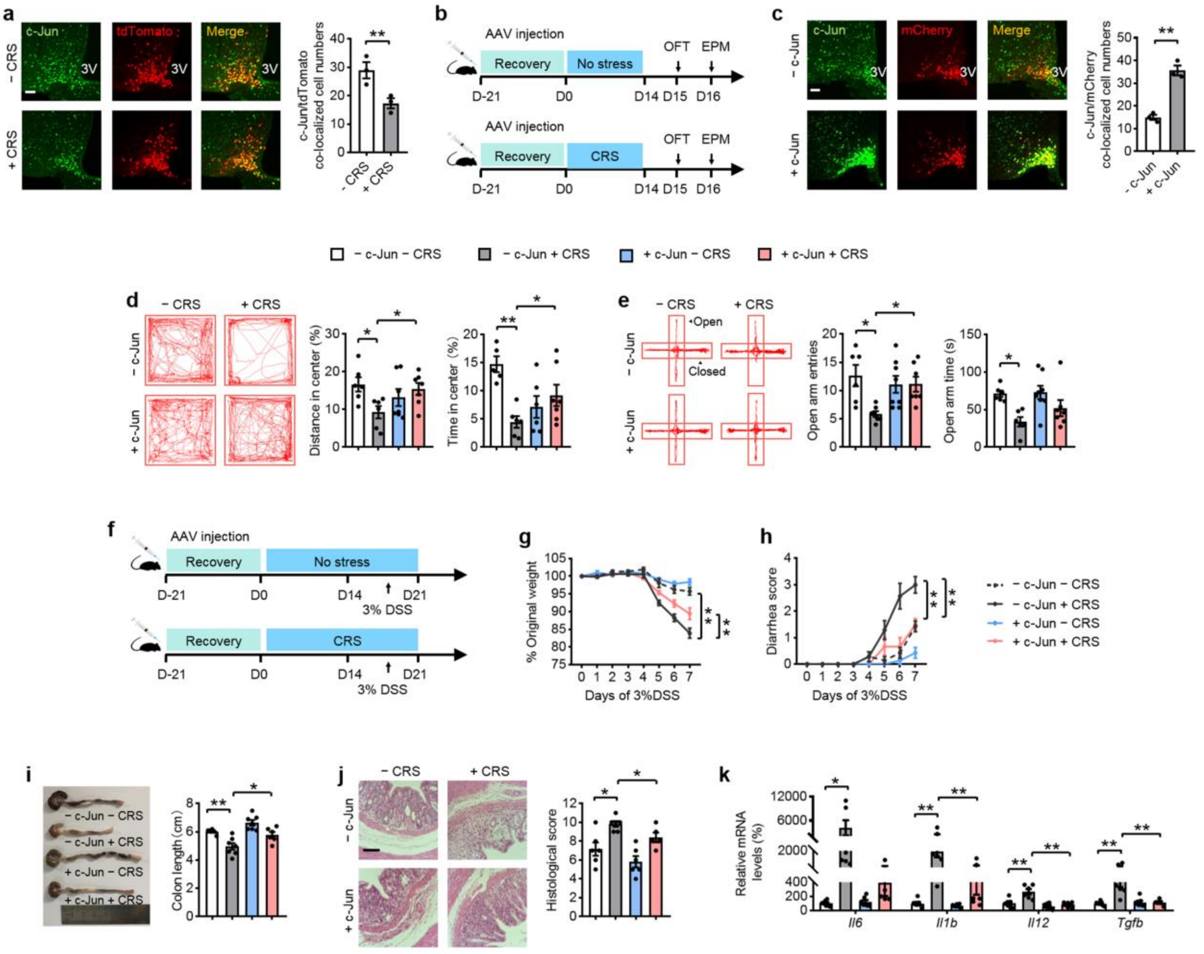

Supplementary Figure 6. Overexpression of c-Jun in AgRP neurons leads to resistance to CRS-induced anxiety-like behaviors and colitis susceptibility. (**a**) IF staining for tdTomato (red), c-Jun (green) and merge (yellow) in ARC sections (left), and quantification of c-Jun and tdTomato colocalized cell numbers (right). Scale bar, 50 μm. (**b**) Experimental timeline for CRS. (**c**) IF staining for mCherry (red), c-Jun

(green) and merge (yellow) in ARC sections (left), and quantification of c-Jun and mCherry colocalized cell numbers (right). Scale bar, 50 μm. (**d**) Representative tracks and statistical results in OF test. (**e**) Representative tracks and statistics in EPM test. (**f**) Experimental timeline for DSS administration. (**g**) Percentage of body weight loss. (**h**) Scores of diarrhea. (**i**) Gross morphology and length of the colon. (**j**) H&E staining and histological scores of the colon tissues. Scale bar, 110 μm. (**k**) qRT-PCR analysis of mRNA expression of inflammatory cytokines (*Il16, Il1b, Il12,* and *Tgfb*) in the distal colonic tissues. Study for **a** was conducted using 14-week-old *AgRP-Cre*-Ai9 mice with (+ CRS) or without (- CRS) 14 days of stress. **c** was conducted using 12- to 14-week-old *AgRP-Cre* mice receiving AAV expressing mCherry (- c-Jun) or c-Jun (+ c-Jun), IF staining performed after 3 weeks from AAV recovery. **d-e** were conducted using – c-Jun mice and + c-Jun mice, both experienced unstressed (- CRS) or stressed (+ CRS) treatment for 14 days. Behavioral tests were performed on day 15 (**d**) and day 16 (**e**). **f-k** were conducted using - c-Jun mice and + c-Jun mice receiving 3% DSS in drinking water for 7 days to induce acute colitis, after stress (+ CRS) or unstress (- CRS). Values are expressed as means ± SEM (n=3-8 per group), with individual data points. Data were analyzed using two-tailed unpaired Student's *t* test (**a, c**). Data were analyzed using two-way ANOVA, followed by Tukey's multiple comparisons test (**d-k**). **P* <0.05, ***P* <0.01.

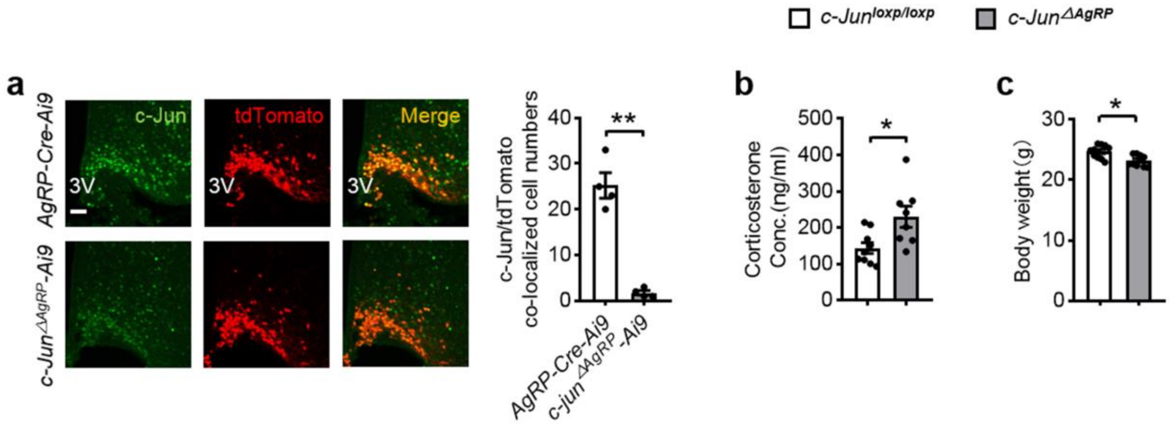

Supplementary Figure 7. Biochemical parameters related to *c-Jun*$^{\Delta AgRP}$ mice. (**a**) IF staining of tdTomato (red), c-Jun (green) and merge (yellow) in ARC sections (left), and quantification of c-Jun and tdTomato colocalized cell numbers (right). Scale bar, 50 µm. (**b**) Serum corticosterone levels. (**c**) Body weight. Study for **a** was conducted using *AgRP-Cre*-Ai9 mice and *c-Jun*$^{\Delta AgRP}$-Ai9 mice. **b-c** were conducted using c-*Jun*$^{loxp/loxp}$ mice or *c-Jun*$^{\Delta AgRP}$ mice. Values are expressed as means ± SEM (n=4-10 per group), with individual data points. Data were analyzed using two-tailed unpaired Student's *t* test. *P <0.05, **P <0.01.

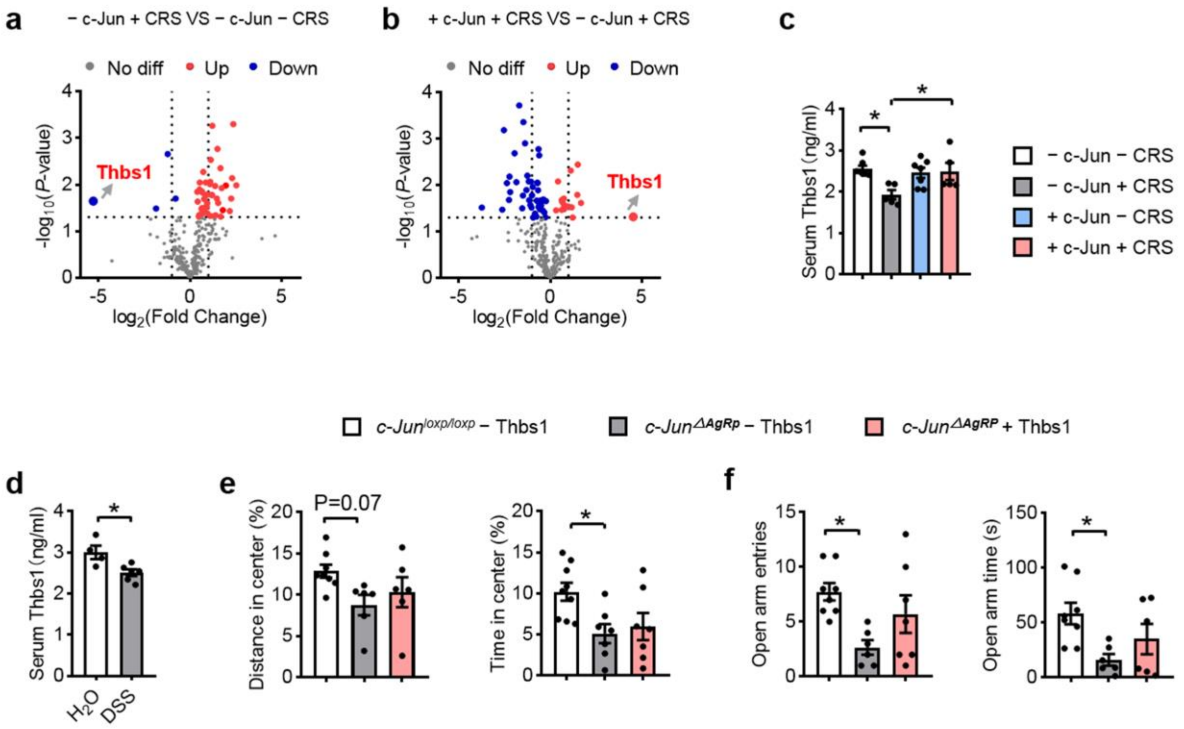

Supplementary Figure 8. Parameters related to *c-Jun*$^{\Delta AgRP}$ mice with thbs1 supplementary. (**a**) Volcano plot of detected proteins between - c-Jun + CRS mice and - c-Jun - CRS after DSS insults; THBS1 is indicated. (**b**) Volcano plot of detected proteins between + c-Jun + CRS and - c-Jun + CRS after DSS insults; THBS1 is indicated. (**c, d**) Serum THBS1 levels. (**e**) Statistical results in OF test. (**f**) Statistics in EPM test. Studies for **a-c** were conducted using - c-Jun - CRS mice, - c-Jun + CRS mice and + c-Jun + CRS mice receiving 3% DSS in drinking water for 7 days to induce acute colitis. **d** was performed using 20- to 22-week-old WT mice with or without 3% DSS administration for 6 days. **e-f** were conducted using 22- to 24-week-old *c-Jun*$^{loxp/loxp}$ mice and *c-Jun*$^{\Delta AgRP}$ mice with (+ thbs1) or without (- thbs1) THBS1 supplementary. Behavioral tests were performed 30 min after single THBS1 injection. Values are expressed as means ± SEM (n=5-9 per group), with individual

data points. Data were analyzed using two-tailed unpaired Student’s *t* test (**d**). Data were analyzed using two-way ANOVA, followed by Tukey’s multiple comparisons test (**c**). Data were analyzed using one-way analysis of variance, followed by Tukey’s multiple comparisons test (**e, f**). *P <0.05, **P <0.01.

Supplementary Tables

Supplementary Table 1. Significant protein expression between -c-Jun + CRS VS -c-Jun - CRS

	P value	FC	log2FC	threshold	S1	S4	S5	S8	S12	S15
Q61805_Lbp	0.010362764	5.753582349	2.5244605	Up	6423600	7659133.315	664247.0021	22250811.9	37435117.01	25162036.74
O09164_Sod3	0.000503851	5.118676461	2.35577082	Up	23505000	18408948.48	23310497.04	98429256.78	107968598.8	127464978.4
P70269_Ctse	0.007294363	4.89526248	2.291386218	Up	12237000	1931409.11	4441647.933	26195898.51	37658006.59	27247208.9
Q02819_Nucb1	0.019923407	4.739932212	2.244866427	Up	13030000	552193.3387	1530851.471	26096126.42	17887257.91	27651423.58
P12246_Apcs	0.036829314	4.47770532	2.162759587	Up	347060000	12025063.92	46451131.97	734862340.3	692832335.9	388176905.7
Q60963_Pla2g7	0.010428459	3.855862922	1.947053764	Up	3835000	2595806.333	1183901.962	7585786.57	9396050.524	12379434.28
Q91ZX7_Lrp1	0.03524588	3.458597913	1.7901873	Up	1615100	763089.6928	439782.8433	4527035.958	2407355.086	2811842.889
P62897_Cycs	0.011902042	3.306130814	1.725143809	Up	3190900	750733.1814	2069979.03	5337806.868	8049266.061	6488103.447
P63017_Hspa8	0.048510012	3.191756581	1.674350629	Up	8335200	4034738.983	4691517.555	26297306.21	16096760.81	12061949.16
O88783_F5	0.004449007	3.149491021	1.655118698	Up	34133000	34600109.73	58468260.63	106671085.7	150236434.3	143712053.8
P16294_F9	0.024840758	2.887881734	1.530011662	Up	16754000	6912510.389	11722085.57	24625714.79	44284174.34	33288190.74
P11859_Agt	0.00171701	2.819601141	1.495491095	Up	124810000	63221020.04	110620851.2	299398053.4	292826722.6	249854380.9
Q9JHH6_Cpb2	0.019811278	2.738527217	1.453400219	Up	81444000	15966753.49	34085662.9	111447061.2	130266413.4	118393040.5
P70663_Sparcl1	0.045553092	2.620834348	1.39002617	Up	33519000	5092929.584	12978449.72	51094759.84	48440843.61	35674234.67
P46412_Gpx3	0.010667772	2.584754868	1.370027466	Up	277970000	117377468.6	259101258.1	506973070.9	678484741.7	506131720
Q61704_Itih3	0.047666312	2.478651547	1.309555469	Up	1405000000	417116763.8	565874686	2413666798	2056045680	1449286223
Q91WP6_Serpina3n	0.00054286	2.327752337	1.21893757	Up	1841400000	1406632955	1444443356	3402555424	3922856629	3597510643
P06728_Apoa4	0.016830111	2.293266889	1.197404265	Up	3513900000	3242081924	3458909480	9015962248	7225607873	7183902113
E9Q414_Apob	0.016558594	2.246340971	1.167576931	Up	898910000	294068069.6	720892322.3	1264881129	1466259183	1568065164
P63260_Actg1	0.002932377	2.172746535	1.119519885	Up	210530000	132466308.8	184521945.9	352048276.5	425128664.1	368986519.5
P98064_Masp1	0.0087125	2.138628069	1.096685604	Up	28246000	20618285.2	42639081.63	69829014.44	58831040.71	67031613.54
Q60590_Orm1	0.046980054	2.134935991	1.094192816	Up	1801400000	519921811.7	999380016.6	2655900350	2540055572	1893529929
Q03734_Serpina3m	0.009719533	2.125225682	1.087616052	Up	2038900000	1553671684	1850019910	4367400612	3030560259	4168774560
P01029_C4b	0.016381044	1.932617735	0.950556306	Up	1753800000	786122004.6	1657263344	2709875416	2895940857	2505738569
Q61147_Cp	0.040923081	1.91584006	0.937977126	Up	4148100000	2359755652	3123370273	7491248425	6361357212	4599282816
P14847_Crp	0.025012019	1.912117499	0.935171179	Up	59197000	29090382.62	43753032.4	93757960.19	89363961.16	69354866.84
P29699_Ahsg	0.029672963	1.881557546	0.911927414	Up	4856000000	3085747642	6329456007	7307242520	9379961143	10164887260
P22599_Serpina1b	0.009020044	1.805635359	0.852506576	Up	1330000000	970774856.2	1373790566	2324035477	2422854030	1888035748
P52480_Pkm	0.016795222	1.782886495	0.834214858	Up	19304000	9845773.423	17815827.13	26495214.79	27399181.5	29839938.65
Q9QXC1_Fetub	0.020449008	1.710363896	0.774303305	Up	832880000	563563958.2	692668133	1048244843	1402344488	1122552565
Q07456_Ambp	0.034691699	1.701606136	0.766897141	Up	673210000	379925166.9	469127137.8	793155411.2	1008759866	788375600.7
P01027_C3	0.031599232	1.674637302	0.743848666	Up	14679000000	8188160567	14439510019	21164768154	23195277343	18115096684
P03953_Cfd	0.005337397	1.649868025	0.722350626	Up	363370000	345822697.8	382571316.1	526665791.8	603617459.8	670983285.8

P19221_F2	0.014623554	1.573229525	0.653729167	Up	1368100000	1310672813	1559038734	2276112161	2506695939	1884242148
P32261_Serpinc1	0.013980131	1.562447162	0.643807402	Up	1145600000	1186544333	1629691524	1891907830	2178413536	2119837823
P06909_Cfh	0.035107423	1.554712584	0.636647897	Up	4512000000	2555807291	3798387518	5717267935	6087099701	5089442169
P03976_	0.043687704	1.549343653	0.631657178	Up	7238600	4764768.435	8913575.633	9941879.965	11767831.84	10697822.73
Q921I1_Tf	0.032219638	1.540993461	0.62386074	Up	31815000000	24505515979	33275740675	51654137795	48644544027	37768563797
P39039_Mbl1	0.019906307	0.57850779	-0.789591707	Down	113410000	147292244.6	165221671.6	88443869.64	85365233.5	72591200.55
Q07968_F13b	0.002194966	0.42808745	-1.224022555	Down	39591000	50253255.71	51082951.61	22133048.12	18818670.67	19377449.97
P29788_Vtn	0.032774966	0.274547332	-1.864873203	Down	136570000	139968476.5	251026653.6	23606730.97	68273588.07	52961279.92
P35441_Thbs1	0.022693544	0.025568924	-5.289464753	Down	141500000	191441421.2	241561149.2	8721062.117	3974224.559	1994125.75

Supplementary Table 2. Significant protein expression between +c-Jun + CRS VS -c-Jun + CRS

	Pvalue	FC	log2FC	threshold	S8	S12	S15	S25	S26	S27
P35441_Thbs1	0.04787214	23.26481839	4.54007802	Up	8721062.117	3974224.559	1994125.75	66786117.6	151821960.5	123138434.2
P98086_C1qa	0.024241786	3.227417781	1.690380343	Up	15038271.08	8365149.308	6392347.737	36149170.68	39213437.95	20800783.23
Q06770_Serpina6	0.003583935	2.836276275	1.503998069	Up	79801316.71	100278170.2	75242796.52	217309987.9	289976725.4	216877821.6
P42703_Lifr	0.016201524	2.798938242	1.484879654	Up	30335622.48	55610212.46	89256880.19	127352631.6	196462280	166566667.8
Q61646_Hp	0.049819739	2.343269165	1.228522682	Up	322967165.4	2625225961	4036390983	4842093627	5733198369	5791468575
Q8BPB5_Efemp1	0.02996872	2.268853528	1.181963475	Up	2142810.105	4819581.268	4786215.753	9227727.078	6625907.204	10802234.45
Q07968_F13b	0.004845746	2.173392476	1.119948723	Up	22133048.12	18818670.67	19377449.97	42925699.33	51081753.65	37111508.46
P06683_C9	0.028629506	1.973082658	0.980451396	Up	72910500	154221877.1	212140758.9	265686030.4	288767066.4	312269109.9
P08607_C4bpa	0.032911748	1.69126885	0.758106014	Up	100467224.4	112870693.5	130095644.2	148913762.2	226693999.8	205230723.7
Q02105_C1qc	0.019376181	1.680816342	0.749162094	Up	35599009.19	19251165.02	29459270.46	45369375.02	50600816.64	45738500.74
P52430_Pon1	0.026311517	1.670435409	0.740224199	Up	161627516.2	142368874.9	112236942.5	194681114.2	273617062.9	226992721.7
P14106_C1qb	0.02275788	1.575875568	0.656153623	Up	28852125.98	17668796.6	21958406.48	37544516.78	37697462.07	32672922.73
P01898_H2-Q10	0.021117946	1.551632021	0.633786454	Up	392971189.9	387355522.5	420016987.5	502834199.7	675662842	683994679.6
P18337_Sell	0.034133612	1.544621624	0.627253474	Up	2047127.034	2060178.732	1347643.891	3215605.415	2542527.635	2667700.15
P32261_Serpinc1	0.002265806	0.658302947	-0.603176439	Down	1891907830	2178413536	2119837823	1386003345	1257947813	1431048878
P11859_Agt	0.027635443	0.651734071	-0.617644676	Down	299398053.4	292826722.6	249854380.9	197301938.2	135481808.7	216027930.6
Q8BND5_Qsox1	0.030060311	0.643719741	-0.635495381	Down	377923595.8	445218248.2	319198782.6	222345367.3	229688881.4	283312964
O88947_F10	0.001676602	0.640126429	-0.643571221	Down	233597541.6	233561711.5	205050650.3	150454709.6	133842915.8	146001699.4
P63260_Actg1	0.012815512	0.630976838	-0.664341046	Down	352048276.5	425128664.1	368986519.5	253807388.4	193886876.9	275508331.1
P01029_C4b	0.009036981	0.619242061	-0.691424627	Down	2709875416	2895940857	2505738569	2035991958	1420471354	1566552628
Q91X72_Hpx	0.008809788	0.611985847	-0.708429806	Down	15680083683	17038506196	13259287983	10299959553	8324697682	9513153282
Q03734_Serpina3m	0.021602577	0.598343422	-0.740954332	Down	4367400612	3030560259	4168774560	2409095371	2352103894	2159680791
Q61147_Cp	0.047618446	0.592167083	-0.755923797	Down	7491248425	6361357212	4599282816	3477202475	3253690066	4195708425
Q9WVJ3_Cpq	0.04114935	0.559850437	-0.83688663	Down	12012723.32	9997409.566	8353508.406	7776567.137	4838050.704	4384479.992
P16294_F9	0.049612691	0.523290009	-0.93431738	Down	24625714.79	44284174.34	33288190.74	16823020.56	16266011.5	20390202.08
Q60590_Orm1	0.010929694	0.514829303	-0.957833923	Down	2655900350	2540055572	1893529929	1168062418	1081766832	1400045812
P27931_Il1r2	0.016575965	0.502071549	-0.994035122	Down	2312653.624	2963250.558	2088311.695	1047455.982	1554230.191	1095677.097
Q61704_Itih3	0.030441067	0.492016275	-1.023222057	Down	2413666798	2056045680	1449286223	1130727810	739501620.5	1042014260
O88783_F5	0.008437467	0.484331782	-1.045932419	Down	106671085.7	150236434.3	143712053.8	64366708.8	57305644.34	72360438.88
P00687_Amy1	0.026854664	0.469485652	-1.090847026	Down	19298539.37	18273993.48	15260739.31	8559174.296	3884761.365	12360527.57
P18242_Ctsd	0.020998759	0.452350565	-1.144486822	Down	2882595.626	3011961.526	4248701.8	1367317.841	1202440.073	2018551.002
P46412_Gpx3	0.008280378	0.441451161	-1.179674258	Down	506973070.9	678484741.7	506131720	232852930	206432211.5	307469021.2
P98064_Masp1	0.006213851	0.41992722	-1.251788786	Down	69829014.44	58831040.71	67031613.54	29364148.61	37318955.86	15493154.02
P29533_Vcam1	0.012679581	0.406718437	-1.297897704	Down	13187580.58	19925738.12	19973960.95	6838166.553	8980254.821	5773154.023
Q91WP6_Serpina3n	0.001260895	0.383888623	-1.381240291	Down	3402555424	3922856629	3597510643	1526144627	948226330.9	1718814792

Q9JHH6_Cpb2	0.000435561	0.36126816	-1.468857986	Down	111447061.2	130266413.4	118393040.5	47826397.5	34124090.34	48144530.22
P70269_Ctse	0.016803765	0.356609013	-1.48758493	Down	26195898.51	37658006.59	27247208.9	4821345.437	11354210.62	16311922.26
Q61207_Psap	0.031450126	0.346803796	-1.527808406	Down	11318571.26	19940499.02	12438692.94	3471135.753	7219810.755	4463603.648
P28798_Grn	0.00019225	0.307759843	-1.700123098	Down	27829870.95	28594814.35	29244735.81	6955011.622	7771278.687	11639317.24
P39061_Col18a1	0.008597943	0.278634261	-1.84355543	Down	6820812.686	9841829.686	6782172.896	1257267.501	3276102.161	1999159.123
P05366_Saa1	0.002052455	0.258483642	-1.951855114	Down	1328964252	1172251588	937477183.6	351117611.9	228547509.6	309180773.5
P12246_Apcs	0.01417923	0.218046743	-2.197290654	Down	734862340.3	692832335.9	388176905.7	139109939.2	73126813.5	183708131.6
Q60963_Pla2g7	0.006530622	0.212112632	-2.237097557	Down	7585786.57	9396050.524	12379434.28	1298278.543	1924235.798	3005382.198
P62897_Cycs	0.020891047	0.209087894	-2.25781856	Down	5337806.868	8049266.061	6488103.447	1529784.661	1262337.704	1363536.409
Q61805_Lbp	0.009012037	0.192717252	-2.375442365	Down	22250811.9	37435117.01	25162036.74	5816773.21	3157600.218	7377293.38
O09164_Sod3	0.00065316	0.171892013	-2.540425579	Down	98429256.78	107968598.8	127464978.4	20971445.17	10872298.08	25544611.51
Q9JM99_Prg4	0.033806506	0.16231137	-2.623164032	Down	17900094.49	45767644.73	43693121.34	3925533.243	11683742.99	1816612.11
P05367_Saa2	0.030562414	0.07476263	-3.741538863	Down	780234107.6	691252919.7	427643432.8	54475524.99	31644289.38	55864174.06

Supplementary Table 3. qRT-PCR primers

Gene	Forward primer 5'-3'	Reverse primer 5'-3'
c-Jun	GCAGAGAGGAAGCGCATGAG	CCTTTTCCGGCACTTGGA
c-Jun-2	GGGAGCATTTGGAGAGTCCC	TTTGCAAAAGTTCGCTCCCG
c-Fos	CCAGTCCTCACCTCTTCCAG	TCCAGCACCAGGTTAATTCC
Il6	CCACGGCCTTCCCTACTTC	TTGGGAGTGGTATCCTCTGTGA
Il6-2	GCCAGAGTCCTTCAGAGAGA	GGTCTTGGTCCTTAGCCACT
Il1b	GCAACTGTTCCTGAACTCAACT	ATCTTTTGGGGTCCGTCAACT
Il12	TGGTTTGCCATCGTTTTGCTG	ACAGGTGAGGTTCACTGTTTCT
Tgfb	AACTTCTGTCTGGGACCCTG	CCGGGTTGTGTTGGTTGTAG
Gapdh	TGTGTCCGTCGTGGATCTGA	CCTGCTTCACCACCTTCTTGAT
