# Supplementary material for: Inhibition of c-Jun in AgRP neurons increases stress-induced anxiety and colitis susceptibility
Source: Commun Biol. 2023 Jan 14;6:50. doi: 10.1038/s42003-023-04425-w (PMC9840628; doi:10.1038/s42003-023-04425-w)
Supplement: Supplementary file 3 — Description of Additional Supplementary Data [file 42003_2023_4425_MOESM3_ESM.docx]

**Description of Additional Supplementary Files**

**File name:** Supplementary Data 1

**Description:** All source data underlying the graphs and charts in the main figures have been uploaded as Supplementary Data in the Excel format.
