# Supplementary material for: Inhibition of c-Jun in AgRP neurons increases stress-induced anxiety and colitis susceptibility
Source: Commun Biol. 2023 Jan 14;6:50. doi: 10.1038/s42003-023-04425-w (PMC9840628; doi:10.1038/s42003-023-04425-w)
Supplement: Supplementary file 5 — Reporting summary [file 42003_2023_4425_MOESM5_ESM.pdf]

nature portfolio

Corresponding author(s): Feifan Guo

Last updated by author(s): Dec 25, 2022

Reporting Summary

Nature Portfolio wishes to improve the reproducibility of the work that we publish. This form provides structure for consistency and transparency in reporting. For further information on Nature Portfolio policies, see our Editorial Policies and the Editorial Policy Checklist.

Statistics

For all statistical analyses, confirm that the following items are present in the figure legend, table legend, main text, or Methods section.

n/a	Confirmed	
☐	☒	The exact sample size (*n*) for each experimental group/condition, given as a discrete number and unit of measurement
☐	☒	A statement on whether measurements were taken from distinct samples or whether the same sample was measured repeatedly
☐	☒	The statistical test(s) used AND whether they are one- or two-sided<br>*Only common tests should be described solely by name; describe more complex techniques in the Methods section.*
☒	☐	A description of all covariates tested
☐	☒	A description of any assumptions or corrections, such as tests of normality and adjustment for multiple comparisons
☐	☒	A full description of the statistical parameters including central tendency (e.g. means) or other basic estimates (e.g. regression coefficient) AND variation (e.g. standard deviation) or associated estimates of uncertainty (e.g. confidence intervals)
☐	☒	For null hypothesis testing, the test statistic (e.g. *F*, *t*, *r*) with confidence intervals, effect sizes, degrees of freedom and *P* value noted<br>*Give P values as exact values whenever suitable.*
☒	☐	For Bayesian analysis, information on the choice of priors and Markov chain Monte Carlo settings
☒	☐	For hierarchical and complex designs, identification of the appropriate level for tests and full reporting of outcomes
☒	☐	Estimates of effect sizes (e.g. Cohen's *d*, Pearson's *r*), indicating how they were calculated

Our web collection on statistics for biologists contains articles on many of the points above.

Software and code

Policy information about availability of computer code

Data collection	1. RT-qPCR: ABI QuantStudio™ 6 Flex Real-Time PCR System;<br>2. H&E images: ECHO Revolve;<br>3. Serum proteomics: Thermo Scientific Q Exactive;<br>4. Fluorescent images: Confocal microscope (Zeiss).
Data analysis	1. Fluorescence density: ZEN2.3 and Image J;<br>2. Serum proteomics: Proteome Discoverer 2.2;<br>3. Anxiety-like behavioral analysis: LabState (AniLab);<br>4. Statistical analysis: GraphPad Prsim 8, Microsoft Excel 2016.

For manuscripts utilizing custom algorithms or software that are central to the research but not yet described in published literature, software must be made available to editors and reviewers. We strongly encourage code deposition in a community repository (e.g. GitHub). See the Nature Portfolio guidelines for submitting code & software for further information.

Data

Policy information about availability of data

All manuscripts must include a data availability statement. This statement should provide the following information, where applicable:

- Accession codes, unique identifiers, or web links for publicly available datasets
- A description of any restrictions on data availability
- For clinical datasets or third party data, please ensure that the statement adheres to our policy

All data presented in this study are available within the Figures and its supplementary information file. The source data underlying the graphs and charts in the main manuscript file are shown in Supplementary Data 1. The raw data of serum proteomics is available in the link: https://doi.org/10.6084/m9.figshare.21776357.v1. Other data that support the study are available from the corresponding author upon reasonable request.

Human research participants

Policy information about studies involving human research participants and Sex and Gender in Research.

Reporting on sex and gender	N/A
Population characteristics	N/A
Recruitment	N/A
Ethics oversight	N/A

Note that full information on the approval of the study protocol must also be provided in the manuscript.

Field-specific reporting

Please select the one below that is the best fit for your research. If you are not sure, read the appropriate sections before making your selection.

☒ Life sciences ☐ Behavioural & social sciences ☐ Ecological, evolutionary & environmental sciences

For a reference copy of the document with all sections, see nature.com/documents/nr-reporting-summary-flat.pdf

Life sciences study design

All studies must disclose on these points even when the disclosure is negative.

Sample size	No sample size calculation was performed, but sample sizes were sufficient to carry out the required experiments with sufficient statistics and standard using t-test for such kind of experiments. All sample numbers were stated in figure legends.
Data exclusions	No data was excluded unless clear technical issues were identified.
Replication	All attempts of replication were successful. All the experiments using mice were supported by at least one additional experiment.
Randomization	Mice were allocated randomly.
Blinding	The investigators were not blinded to allocation during experiments and data analysis, as objective quantitative assays were used when we generated the data.

Reporting for specific materials, systems and methods

We require information from authors about some types of materials, experimental systems and methods used in many studies. Here, indicate whether each material, system or method listed is relevant to your study. If you are not sure if a list item applies to your research, read the appropriate section before selecting a response.

Materials & experimental systems

n/a	Involved in the study	
☐	☒	Antibodies
☒	☐	Eukaryotic cell lines
☒	☐	Palaeontology and archaeology
☐	☒	Animals and other organisms
☒	☐	Clinical data
☒	☐	Dual use research of concern

Methods

n/a	Involved in the study	
☒	☐	ChIP-seq
☒	☐	Flow cytometry
☒	☐	MRI-based neuroimaging

Antibodies

Antibodies used	anti-c-Jun (Cat. No. 9165, 1:1000, Cell Signaling Technology, MA, USA);<br>anti-c-Fos (Cat. No. 2250, 1:1000, Cell Signaling Technology, MA, USA or Cat. No. sc-52, 1:500, Santa Cruz Biotechnology, CA, USA);<br>Donkey anti-Rabbit IgG Alexa Fluor™ 488 (1:1000; A-21206; Thermo Fisher).
Validation	All antibodies used in this work were purchased from companies, and validated by the manufacturers and by extensive use in published manuscripts.

Animals and other research organisms

Policy information about studies involving animals; ARRIVE guidelines recommended for reporting animal research, and Sex and Gender in Research

Laboratory animals	Adult C57BL/6J wild-type mice were purchased from Shanghai Laboratory Animal Co., Ltd. (Shanghai, China);<br>c-Jun loxp/loxp mice were a kind gift from Dr. Erwin F. Wagner (Cancer Cell Biology Program, Spanish National Cancer Research Center) ;<br>AgRP-irs-Cre mice and Ai9 mice were obtained from Jackson Laboratory (Bar Harbor, ME, USA)
Wild animals	The study did not involve wild animals.
Reporting on sex	All mice used in this study were male.
Field-collected samples	The study did not involve samples collected from the field.
Ethics oversight	All mice experiments were performed in accordance with the procedures of the Institutional Animal Care and Use Committee of the Shanghai Institute of Nutrition and Health, Chinese Academy of Sciences.

Note that full information on the approval of the study protocol must also be provided in the manuscript.
